# Editorial: Postoperative care: from pain management to delirium

**DOI:** 10.3389/fmed.2023.1179358

**Published:** 2023-06-15

**Authors:** Zhongheng Zhang

**Affiliations:** Department of Emergency Medicine, Key Laboratory of Precision Medicine in Diagnosis and Monitoring Research of Zhejiang Province, Sir Run Run Shaw Hospital, Zhejiang University School of Medicine, Hangzhou, China

**Keywords:** postoperative care, pain, delirium, analgesia, sedation: propofol

Postoperative care is important for the success of surgical operations. Pain and delirium are among the most important adverse events especially for the elderly (1). It has been reported that postoperative delirium (POD) has a negative impact on prognosis, length of stay and the burden of care. Many efforts have been made to predict the POD in the literature (2). Many novel biomarkers such as the changes in plasma tau and neurofilament light (NfL) are found to be associated with increased risk of POD (3, 4). Pain management is directly related to the development of postoperative delirium. And thus, improved control of pain can not only improve the patients' comfort but also reduce the risk of POD. Thus, the management of pain and delirium are usually inseparable. For some elderly patients with major operation, appropriate management of pain and delirium are also of vital importance to the postoperative rehabilitation (5). In this regard, I launched a special topic in Frontiers in Medicine to report most updated advances in postoperative care of pain and delirium management.

A total of 15 articles are finally published after rigorous peer review process. Zheng et al. explored nutritional status and postoperative pain outcome in elderly patients. They found that high nutritional risk/malnutrition was associated with poor postoperative pain outcomes (i.e. inadequate analgesia, cumulative consumption of analgesics) in older patients following gastrointestinal surgery, and further proposed a cut-off value of 88 for geriatric nutritional risk index (GNRI) for clinical utility. In a randomized controlled trial, Xu et al. compared dexmedetomidine combined with butorphanol or sufentanil for the prevention of postoperative nausea and vomiting (PONV) in patients undergoing microvascular decompression. The authors tested the analgesics in this special population because patients undergoing microvascular decompression are often accompanied with high risk of post-operative nausea and vomiting. They concluded that butorphanol combined with dexmedetomidine could reduce early PONV and the number of patients requiring rescue antiemetics. Acupuncture is an important component in the traditional Chinese medicine and many studies have proven its efficacy in alleviating symptoms such as postoperative delirium (6). In this special issue, Fan et al. compared transcutaneous electrical acupoint stimulation combined with auricular acupressure vs. usual care on the incidence of postoperative delirium among older patients undergoing major abdominal surgery. The postoperative delirium is significantly reduced by the use of this intervention [19/105 (18.1%) vs. 8/105 (7.6%), difference, −10.5% (95% CI, −1.5% to −19.4%); hazard ratio, 0.41 [95% CI, 0.18 to 0.95); *P* = 0.023]. In addition to clinical investigations, we also published experimental studies. Mu et al. developed an animal model of postoperative delirium and found that interleukin-6 played an pivotal role in the pathological process.

## Author contributions

ZZ design and drafted this editorial.
